# The Institutional Treatment of Consumption

**Published:** 1916-06-17

**Authors:** 


### THE INSTITUTIONAL TREATMENT OF CONSUMPTION.

### IV.—Sanatorium Buildings and Equipment.*

It is a common error to imagine that a warm climate is necessarily beneficial to consumptives; the resorts which have been most successful in the treatment of this disease are mostly those which are cold during some part of the twenty-four hours. Persons in this country domiciled in the North do not, as a rule, do well on the South Coast. A number of patients at Brompton were once sent to Madeira, but the results were on the whole not encouraging. However, in England the climate must be taken as it comes, and fortunately for the working-class sanatoria now springing up everywhere, our climate is not so bad as it is painted.

So many considerations are involved in the question of choice of site that it will not be profitable to go deeply into the matter. Dry soil, sheltered situation, bracing unpolluted air, and rising ground with a southerly aspect, at once occur to the mind as desiderata. A sanatorium should not be very near a high-road on account of the dust which will rise in dry weather, and while a railway station should be reasonably accessible, the institution ought to be beyond the range of the sound of railway traffic. A treeless site is a great disadvantage, but trees must not be allowed crowded around the buildings. A sheltered walk for windy weather is of importance.

### Essentials *v.* Non-Essentials.

The best practical ideas on the construction of a sanatorium would be gained by visiting a large number of institutions both in this country and abroad. The observer might, unless he carefully co-ordinated his notes, have some difficulty in arriving at a judgment as to the best means of securing some essential feature and in differentiating the ingeniously constructed non-essential item from the really necessary feature. The greatest variety exists in sanatorium buildings, and it would serve no useful purpose to attempt to describe in detail a model type. Cost is a consideration which has a great influence, and in fact it should have had a greater restraint than it did in those instances where sanatoria for the working classes have cost over £500 a bed. The ideal of the £100-a-bed institution has not yet been entirely satisfactorily achieved; taking into account the expense of preparing, draining, and fencing the land, it is often impossible to keep down the total cost, including equipment, below £200. The cost is seriously affected by the number of beds provided and the character of construction.

Large sums spent on mere bricks and mortar are now recognised as extravagant. The open-air ward is coming into favour; it is economically constructed, and for properly selected cases is greatly to be preferred. But in the same grounds there must also be provided either small wards or single rooms for those patients who are unfit to endure the rigours of the open-air ward until they have settled down and become acclimatised; and also for patients who may need transferring as "hospital" cases or on account of acute exacerbations.

A sanatorium in the Midlands recently constructed on such lines cost for the buildings alone about £120 per bed, including an advance in charges owing to the war. The total cost of the land, road-making, drainage, and fully-equipped buildings was £220 per bed. Our readers will recall that the open-air temporary military hospital at Cambridge (1st Eastern General Hospital) was said to have been erected at a cost of only £20 per bed, exclusive of accommodation for nurses and medical staff, but this hospital contained over 1,500 beds and was by no means constructed on sanatorium lines. Although details are wanting as to what exactly is included in the total whence this extraordinary figure is derived, the statement is an interesting one to bear in mind.

### Some Principles of Design.

High buildings are obviously unsuited for a sanatorium, and one-storey buildings have many advantages; in fact, these are now the usual pattern. For one thing it should always be possible to draw patients' beds into the open air; the space may be called a verandah or a *Liegehalle*, or may be part and parcel of the ward. If this can be done above the ground floor it usually means that the ward below is darkened by an overhanging balcony. Then, again, the inhabitants of the ground floor are apt to be disturbed by the noises made by their fellow-patients overhead. On the score of expense free sanatoria for the working classes will be constructed with wards for, say, a dozen beds or so, and not on the plan of cubicles or separate rooms. The use of common rooms should not be encouraged to any extent, as they are apt to tempt patients to stay indoors when they should be outside, or if it is raining, in the protected open-air shelter. Verandahs placed due south may be too hot, particularly when roofed with glass, for patients even in this country in summer, and if placed south of a ward will darken it and may interfere with ventilation. A verandah requires to be ventilated, and should therefore have a roof in two overlapping sections or have open ends.

As in an operating theatre there should be an absence of dust-traps; rounded corners, no ledges projecting, and a smooth washable surface for the walls are necessary. Floors may be constructed of boards grooved and tongued and kept well waxed. Various compositions of a plastic nature are available for floors and resemble artificial stone; these materials make a rather noisy floor, and this disadvantage may suggest slippers of felt or strips of carpet.

Well-painted cement walls are probably as good as anything, though a first-class washable distemper is almost as good, and is certainly preferable

* The previous articles appeared on May 6 and 20, and June 3, 1916.

to washable paper, which is not uncommon abroad. The colouring of the walls is nowadays considered as being of special importance from the point of view of the patient. Bright colours with vivid, not to say startling, contrasts are said to have a cheering influence on the minds of the patients. Certainly, judging from the effect produced by the brilliantly enamelled walls of a new sanatorium at Lübeck, there may be something in the idea. But as yet we have no direct evidence as to the favourable opinions of those who are daily confronted with vivid yellow, blue, green, and red walls.

Furniture in a sanatorium should naturally be plain, light, and easily cleaned. Upholstery should be practically eliminated, but cushions with washable covers are certainly allowable. Plain iron bedsteads with a simple pattern of locker must suffice for the patients' use in the wards; cupboards for the greater part of their belongings and for boots and hats being provided outside the ward. Cupboards also must be ventilated and capable of being easily cleaned.

In order to protect from rain beds on a verandah or in a three-sided open-air ward, some sort of waterproof covering is necessary for the foot end, for in this country we cannot afford to withdraw patients indoors every time it rains. An apparently satisfactory device is used at the Derbyshire County Sanatorium in the form of a canopy which is attached to the end of the bed. This can be drawn up easily and speedily to cover like a tent the lower half of the bed, and when not in use is neatly folded up out of the way.

The above-mentioned sanatorium is one of the most recent additions to the existing accommodation for tuberculous patients, and it may be interesting to glance for a moment at some of the details of the cost of its furnishing and equipment. The number of patients provided for is one hundred; there are about ten nurses' bedrooms and a domestic staff of ten.

| | |
|---|---|
| Bedsteads, 125 (sixty with canopies) | £224 |
| Mattresses, 125 | 210 |
| Pillows | 56 |
| Blankets, 625 | 240 |
| Rest chairs | 100 |
| Rugs for rest chairs | 28 |
| Furniture | 551 |
| Lockers | 67 |
| Cupboards | 130 |
| Crockery and glass | 79 |
| Cutlery | 34 |
| Kitchen and ironmongery | 114 |
| Fire appliances | 78 |
| Food trolleys (2) electrically heated | 50 |
| Linen | 233 |
| X-ray installation | 200 |
| Medical equipment | 175 |
| Out-door tools | 30 |
| Total | £2,599 |

Having regard to the mode of life of patients undergoing true sanatorium treatment, it will be surmised that the heating arrangements required need not be elaborate. For a considerable portion of the year artificial heating can be dispensed with, and, indeed, some sanatoria have found it possible to manage without heating even during the winter. But this entails unnecessary hardship both on the patients and on the staff, and although those patients who are in bed may be kept warm, those who are up and about but not yet on hard work or exercise will suffer severely, and even the workers are not always at work.

Hot pipes in open-air wards may be dispensed with, it is true, but access to some source of heat should be provided for patients while dressing, while at meals, and at the evening recreation time. Dining-rooms need not be warmed throughout, which would entail closed windows; all that is necessary in practice is to run some hot pipes under it in a closed channel, care being taken that the arrangement is one which can be easily cleansed from dust, etc. This method has been criticised, but has proved sufficient and is certainly economical. The patients are comfortable during meals, and the warmth is far more equally apportioned than it would be if radiators were placed at intervals around the hall, while free access of fresh air continues.

